# Down-regulated HSA_circ_0003528 inhibits hepatocellular carcinoma aggressiveness via the miR-212-3p/*XIAP* axis

**DOI:** 10.1080/21655979.2022.2066046

**Published:** 2022-04-29

**Authors:** Qi Liu, Xin Xu, Wei Sun

**Affiliations:** a Department of Gastroenterology, The Second Affiliated Hospital, Hengyang Medical School, University of South China, Hengyang, Hunan, China; b Department of Blood Transfusion, The Second Affiliated Hospital, Hengyang Medical School, University of South China, Hengyang, Hunan, China; c Department of Gastroenterology, The Fourth Affiliated Hospital of Inner Mongolia Medical University, Baotou, Qingshan, China

**Keywords:** Hepatocellular carcinoma, circ_0003528, *XIAP*, apoptosis

## Abstract

Hepatocellular carcinoma (HCC) is characterized by a high mortality rate. Dysregulated circular RNAs (circRNAs) play a vital role in HCC. We aimed to study the role of circ_0003528 in HCC and its fundamental molecular mechanisms. HSA_circ_0003528 was identified through bioinformatics dataset analysis. The binding sites between mRNA and miRNA were predicted using online bioinformatics tools. The interaction between miR-212-3p and X-linked inhibitor of apoptosis protein (*XIAP*) or circ_0003528 was confirmed through the luciferase reporter assay. RT-qPCR and western blot assays were used to analyze the expression of all miRNAs/mRNAs and proteins. Cellular functions were evaluated using the MTT and TUNEL assays. A xenograft model was established to evaluate the function of circ_0003528 *in vivo*. Circ_0003528 was dramatically overexpressed in HepG2 and HUH7 cells. However, knockdown of circ_0003528 suppressed the aggressiveness of HCC cells and tumor growth both *in vitro* and *in vivo*. Furthermore, binding of miR-212-3p to circ_0003528 and *XIAP* was verified. Downregulation of miR-212-3p abrogated the effects of si-circ_0003528 on cell viability and apoptosis, and upregulation of *XIAP* antagonized the functions of the miR-212-3p mimic in HCC cells. circ_0003528 contributes to the development of HCC *in vitro* and *in vivo* via the miR-212-3p/*XIAP* axis. Hence, circ_0003528 knockdown may be a potential therapeutic strategy for HCC treatment.

## Graphical abstract

**Figure:**
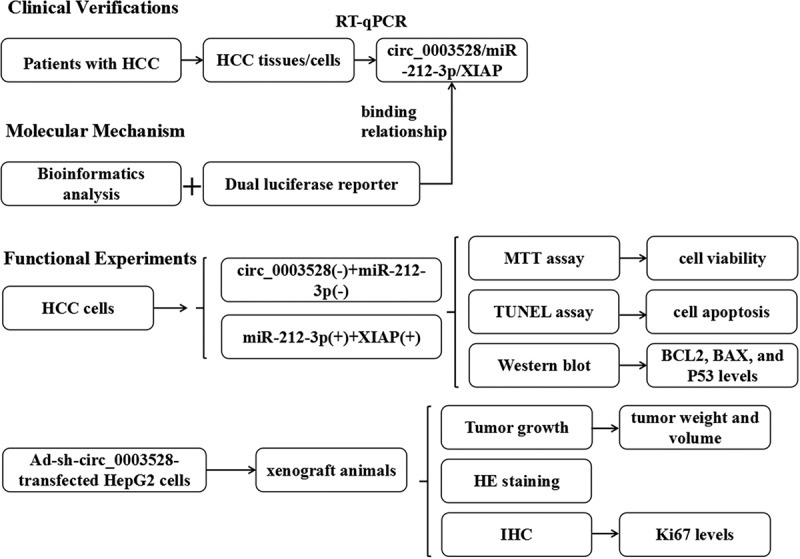

## Highlights

- circ_0003528 was overexpressed in the HCC tissues and cell lines.
- Knockdown of circ_0003528 inhibited the cell viability, promoted cell apoptosis *in vitro*, and suppressed tumor growth *in vivo*.
- The knockdown of CCAT2 suppressed the aggressiveness of HCC cells via the miR-212-3p/XIAP axis.

## Introduction

Hepatocellular carcinoma (HCC) is a hematology-rich malignant tumor that originates from liver cells [1]. Currently, HCC is the second leading cause of cancer-related deaths worldwide [2,3]. Transcatheter arterial chemoembolization is recognized as one of the most commonly used nonsurgical treatment methods for HCC [4]. However, the statistical results of clinical data showed that patients who received transcatheter arterial chemoembolization still suffered from intrahepatic vascular invasion or distant metastasis and had a poor prognosis [5–7]. Therefore, the development of a promising and efficient strategy for the treatment of HCC is urgently needed.

Circular RNAs (circRNAs), a novel subtype of non-coding RNAs, are diffusely present in nearly all eukaryotes [8,9]. Evidence suggests that circRNAs can interact with a vital non-coding RNAs sub-type, the microRNAs (miRNAs), to regulate target genes of miRNAs and thus participate in the regulation of various diseases, including human cancer [10]. In the last three decades, several circRNAs have been verified to function as essential regulators of HCC tumorigenesis through competing endogenous RNA mechanisms [11–14]. For example, HSA_circ_0006091 has been reported as a novel molecular marker for HCC diagnosis [15]. circ_0001445 and circRNA-5692 inhibit the aggressiveness of HCC cells in diverse ways [12, Liu 13], whereas circMET and circ_KIAA1429 accelerate HCC progression [16,17]. However, the regulatory network of circRNAs in HCC requires further investigation. circ_0003528 has been identified as a dysregulated circRNA in lung adenocarcinoma through circRNA-miRNA-mRNA networks [18]. circ_003528 is located on the long arm of chromosome 5, which has been reported to be the rallying point of proposed tumor suppressor genes in HCC without cirrhosis [19]. Recently, by analyzing microarray datasets, circ_0003528 was found to be aberrantly expressed in HCC tissues. In this study, circ_0003528 was identified as a novel circRNA in HCC using RNA sequencing analysis. The role and function of circ_0003528 in HCC were studied both *in vitro* and *in vivo*.

In this study, the potential role of circ_0003528 and underlying mechanism in HCC was investigated. The effect of circ_0003528 on the phenotype of HCC cell lines was determined by functional studies.

## Materials and methods

### circRNAs expression profile analysis

To identify the potential functional circRNAs in HCC, the GEO database (https://www.ncbi.nlm.nih.gov/geo/) was used to acquire the dataset (GSE97332) for differential expression analysis [20]. Based on the GPL19978 Agilent-069978 Arraystar Human CircRNA microarray Version 1.0 platform (CA, USA), seven pairs of HCC and matched non-tumor liver tissue were analyzed for differential expression using an online tool GEO2R (http://www.ncbi.nlm.nih.gov/geo/geo2r/). R language was used to analyze the dataset, then limma package was applied to identify all differentially expressed circRNAs. Finally differentially expressed circRNAs in this study were screened under the standard of *P* < 0.05 and |log2FC| ≥ 1.5, and the volcano plot.

## Clinical HCC samples

In total, 57 pairs of HCC and adjacent normal tissues were surgically removed from patients undergoing primary HCC surgery in Hengyang Medical School, University of South China. Written informed consent was obtained from all patients, and the study was approved by the ethics committee of Hengyang Medical School, University of South China and was conducted in accordance with the Declaration of Helsinki.

## Cell culture

Two HCC cell lines, HepG2 and HUH7, were cultured in Dulbecco’s modified Eagle medium (Thermo Fisher, CA, USA), and normal hepatocytes THLE-2 were cultivated in RPMI-1640 medium (Thermo Fisher, CA, USA) containing 10% FBS (Sigma-Aldrich, NJ, USA) at 37°C. All cell lines were purchased from the ATCC (CA, USA).

## RNA interference

Cell transfection was performed using Lipofectamine 3000 (Life Technologies, USA) according to the manufacturer’s instructions, and RT-qPCR was used to detect the transfection efficiency [11]. Briefly, the HCC cell lines mentioned above were seeded into 6-well plates and transfected with oligonucleotide (50 nM miRNA/20 nM siRNA) or plasmid (2 μg) at a cell concentration of 5 × 10^5^ cells per well. After transfection for 24 h, all cells were collected for subsequent experimentation, followed by replacement with fresh complete medium. si-circ_0003528 1#, si-circ_0003528 2#, miR-212-3p mimic, mimic nc, miR-212-3p inhibitor, inhibitor nc, X-linked inhibitor of apoptosis protein (*XIAP*), and the control vector were designed by Miaoling Biotech (Wuhan, China).

## RT-qPCR

Total RNA from HepG2, HUH7, and THLE-2 cells was distilled using TRIzol reagent (Qiagen GmbH, Hilden, Germany). All mRNA and miRNA were reverse-transcribed using the First Strand cDNA Synthesis Kit (Merck, NJ, USA), and PCR was performed on an ARIAMX real-time PCR system (Agilent, CA, USA). The expression levels of circ_0003528/*XIAP* (relative to *GAPDH*) and miR-212-3p (relative to *U6*) were calculated using the 2^−ΔΔCt^ method [11]. The qRT-PCR conditions consisted of an initial denaturation at 95°C for 30s, followed by 40 cycles of denaturation at 95°C for 5s, and annealing at 60°C for 30s. The conditions of the melting curve analysis were one cycle of denaturation at 95°C for 10s, followed by an increase in temperature from 65°C to 95°C at a rate of 0.5°C/s. All primer sequences were synthesized by Realgene (Nanjing, China): circ_0003528 (Forward, 5ʹ-GTAACCAGCAGCCTGGACTC-3ʹ; Reverse, 5ʹ-GCAACTTGCTGACCAGAACA-3ʹ) [21,22]; miR-212-3p (Forward, 5ʹ-CGGCGGTAACAGTCTCCAGTC-3ʹ; Reverse, 5ʹ-GTGCAGGGTCCGAGGT-3ʹ); *XIAP* (Forward, 5ʹ-GCTCCACGAGTCCTACTGTG-3ʹ; Reverse, 5ʹ-GTTCACTGCGACAGACATCTC-3ʹ); *GAPDH* (For-ward, 5ʹ-GGAGCGAGATCCCTCCAAAAT-3ʹ; Reverse, 5ʹ-GGCTGTTGTCATACTTCTCATGG-3ʹ), *U6* (Forward, 5ʹ-GATTATCGGGACCATTCCACTG-3ʹ; Reverse, 5ʹ-GATCTGGTTCCCAATGACTGTG-3ʹ). Each experiment was performed at least three times.

## Fluorescence in situ hybridization (FISH)

Hybridization was performed overnight with circ_0003528 probes. Specimens were analyzed using a Nikon inverted fluorescence microscope. The circ_0003528 probe for FISH is 5ʹ-TGTATATCCTGTTTAAGGAGAGCC-3’. This assay was repeated three times.

## MTT assay

For the MTT assay, cells were adjusted to 1 × 10^5^ cells/mL and seeded in 96-well plates (100 μL/well) [23–25]. Ten microliters of MTT reagent (Solarbio, Beijing, China) were added to each well of the plate at six time points. The cells were cultivated in the incubator at 37°C for 4 h and absorbance values were measured using a microplate reader (Spectra Max 250 Spectrophotometer; Molecular Devices, CA, USA) at a wavelength of 490 nm.

## TUNEL assay

A TUNEL kit (40308ES20; YEASEN, Shanghai, China) was used to measure cell death based on the manufacturer’s protocol. Briefly, HCC cells were mixed with phosphate buffered saline (PBS) containing 4% formaldehyde for 15 min, followed by permeabilization with 0.2% Triton X-100 in PBS for another 10 min under the same conditions. After washing twice with PBS, the HCC cells were co-cultured with TdT mixture at 37°C for 0.5 h. Finally, 4ʹ,6-diamidino-2-phenylindole (DAPI) was used to dye the nuclei again [26]. Apoptotic cells were observed and analyzed using a fluorescence microscope (90i; Nikon, Tokyo, Japan).

## Western blot assay

The antibodies used were anti-Bcl2 (ab182858, 1: 2000), anti-Bax (ab182733, 1: 2000), anti-p53 (ab32389, 1: 10000), anti-GAPDH (ab8245, 1: 500), and secondary antibody (ab96899, 1: 1000). All antibodies were purchased from Abcam (Cambridge, UK). RIPA buffer (Sigma-Aldrich, NJ, USA) was used to obtain total protein from all cells, followed by quantification of proteins using a BCA kit (Beyotime, Jiangsu, China). Subsequently, 20 µg proteins was isolated using a 10% sodium dodecyl sulfate–polyacrylamide gel and then electrotransferred to polyvinylidene fluoride membranes, blocked with 5% skimmed milk for 1 h, and then incubated overnight with primary antibodies at 4°C [27]. Next day, the membranes were incubated with secondary antibody at 37°C for 1.5 h. Finally, protein bands were visualized using an ECL system (Thermo Fisher Scientific, USA).

## Luciferase reporter assay

The wild-type or mutant miR-212-3p binding sequence of circ_0003528 and the 3-UTR of *XIAP* were inserted into the psi-CHECK-2 plasmid to construct luciferase reporter plasmids. After seeding into 24-well plates, HCC cells were co-transfected with luciferase reporter and miR-212-3p mimic or miR-212-3p mimic nc using Lipofectamine 3000 (Invitrogen, USA). Firefly and Renilla luciferase fluorescence signals were detected using a dual-luciferase reporter assay kit (Promega, USA) [27].

## *In vivo* tumorigenesis and metastasis assay

Eight mice (4-week-old nude male BALB/c mice), purchased from the Animal Center of Nanjing Medical University, were randomly divided into two groups. Ad-sh-circ_0003528-transfected HepG2 cells and Ad-sh-nc-transfected HepG2 cells were suspended in Dulbecco’s modified Eagle medium at a final concentration of 3 × 10^6^ cells. The two groups of cells were then injected into the backs of the mice [25]. Thirty days later, mice were sacrificed and the tumors were dissected to measure the volumes (calculated as [width × length × height]/2) and weights of the tumors. All animal experiments met the requirements of laboratory animal welfare and ethics and were approved by the Hengyang Medical School, University of South China (No.20190225).

## HE staining and immunohistochemistry (IHC) staining

The transplanted tumor tissues were fixed in 4% formaldehyde solution, embedded in paraffin, and sectionalized in 4 μm thickness. HE staining and IHC staining were performed.

HE staining: The sections were stained with hematoxylin for 5 min, washed with deionized water, followed by differentiation with hydrochloric acid and ethanol, washed with deionized water and dyed with eosin staining solution for 3 min, dehydrated with ethanol, transparent with xylene, and sealed with neutral gum. The stained sections were observed under an optical microscope.

IHC staining: The sections were dewaxed to water and incubated in 3% H_2_O_2_ solution for 10 min. Subsequently, the mouse anti-human Ki67 monoclonal antibody in PBS buffer containing slices was incubated overnight at 4°C. On the second day, antibody HRP-labeled Goat anti-mouse IgG was added to the buffer and incubated at 37°C for 20 min. Then DAB was used for staining, hematoxylin was used for contrast staining, ethanol dehydration, xylene transparent, neutral gum sealed.

## Statistical analysis

SPSS software (version 19.0; IBM, Armonk, NY, USA) was used to analyze all data, which are presented as means ± standard deviation. The Student’s *t*-test and one-way ANOVA were used for analysis of differences. Statistical significance was set at *p* < 0.05.

## Results

### Circ_0003528 is upregulated in HCC

**Figure 1.**
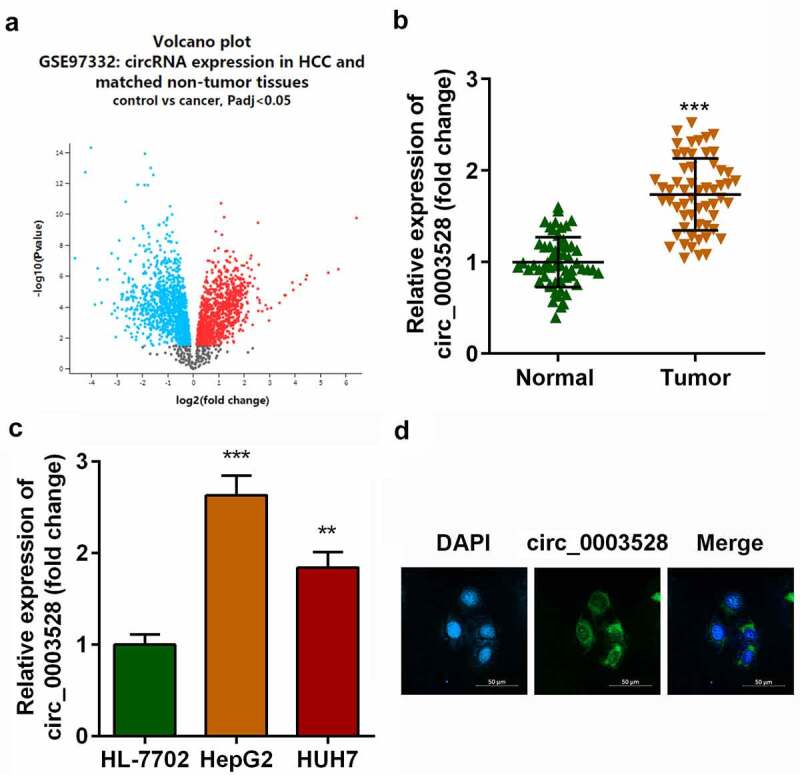
Circ_0003528 is upregulated in HCC.(a) Volcano map of differentially expressed circRNAs in HCC clinical samples. Expression of circ_0003528 in (b) HCC tissues and (c) cells. (d) Location of circ_0003528 in HCC cells accessed from FISH. **p< 0.01, ***p< 0.001.

The microarray dataset GSE97332 was used to screen for differentially expressed circRNAs in patients with HCC. A total of 453 upregulated and 439 down-regulated differentially expressed circRNAs were identified (Figure 1a), among which circ_0003528 was remarkably upregulated in HCC tissues (Figure 1b). In addition, as shown in Figure 1c, circ_0003528 was overexpressed in HepG2 and HUH7 cells compared with that in HL-7702 cells (Figure 1c). Furthermore, FISH experiments were performed, which demonstrated the cytoplasmic enrichment of circ_0003528 (Figure 1d).

## Knockdown of circ_0003528 suppresses the aggressiveness of HCC cells *in vitro*

**Figure 2.**
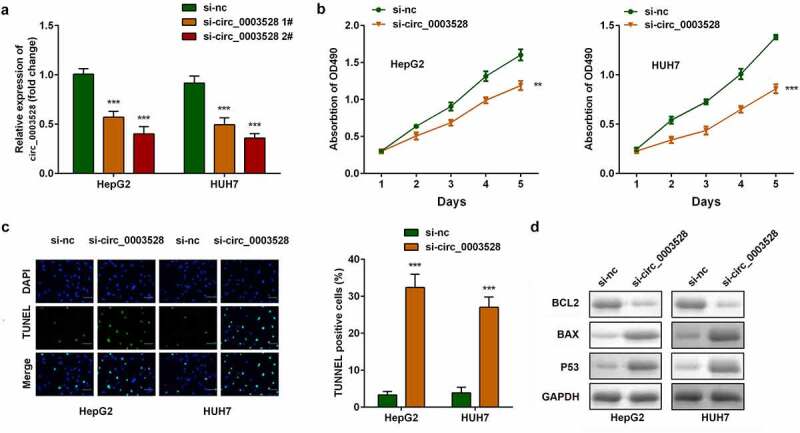
Knockdown of circ_0003528 suppresses the malignant behavior of HCC cells. (a) circ_0003528 expression was determined using RT-qPCR. (b) Cell viability was measured using the MTT assay. (c) The TUNEL assay was performed to determine cell apoptosis. (d) Expression of Bcl2, Bax, and p53 proteins.

Then the role of circ_0003528 in modulating HCC aggressive behaviors was investigated. The expression of circ_0003528 in the silenced groups was approximately two-fold lower than that in the control group, and was highly noticeable in the si-circ_0003528 2# group (Figure 2a). In both HepG2 and HUH7 cells, cell viability was suppressed, and cell apoptosis was clearly facilitated in the down-regulated circ_0003528 groups (Figure 2b-c). Furthermore, the expression of apoptosis-related proteins, including Bcl2, Bax, and p53, was measured to verify the suppression of apoptosis (Figure 2d).

## miR-212-3p was a target of circ_0003528

**Figure 3.**
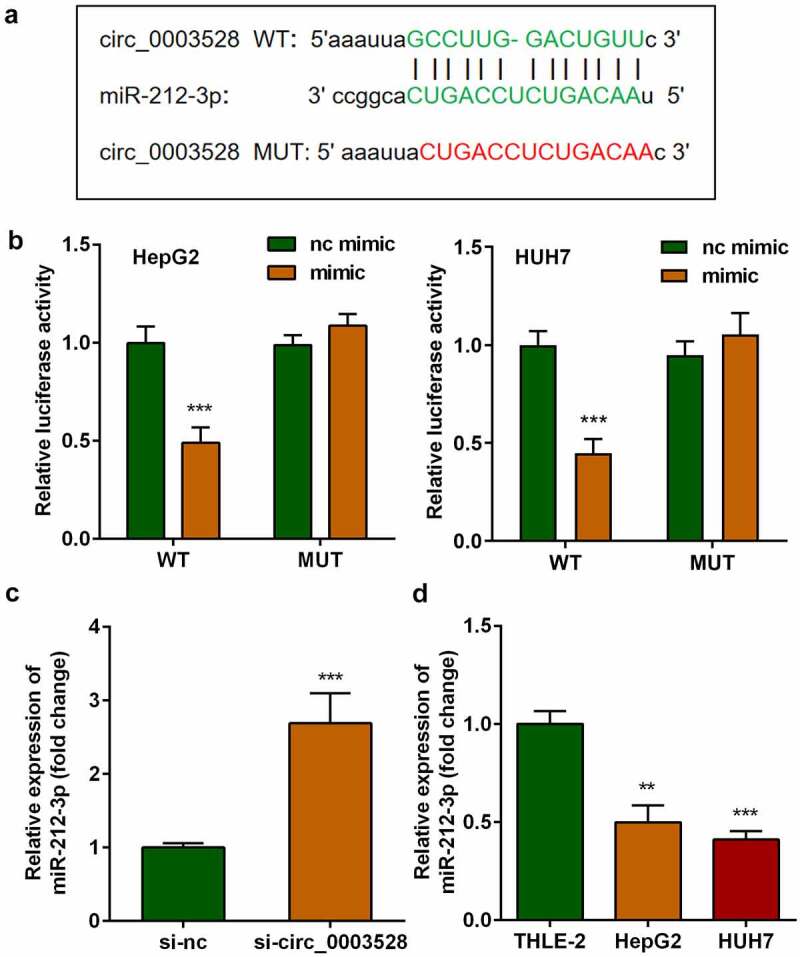
miR-212-3p functions as a target of circ_0003528. (a) Binding sites between miR-212-3p and circ_0003528. (b) Dual-luciferase reporter assay was performed to confirm the interaction between miR-212-3p and circ_0003528 in HCC cells. (c-d) miR-212-3p expression.

Afterward, whether circ_0003528 regulated cellular functions of HCC cells by binding miRNAs was studied. As shown in Figure 3a, the binding sites between miR-212-3p and circ_0003528 predicted by Starbase v.3.0 (http://starbase.sysu.edu.cn/) illustrated that miR-212-3p could potentially interact with circ_0003528. Next, the results of the dual-luciferase reporter assay indicated that co-transfection with miR-212-3p mimic and 3'UTR wildtype of circ_0003528 remarkably decreased luciferase activity. Finally, miR-212-3p expression levels were increased by si-circ_0003528 and down-regulated in HCC cells (Figure 3c-d).

## Downregulation of miR-212-3p abrogates the function of si-circ_0003528 on regulating cell viability and apoptosis of HCC cell lines

**Figure 4.**
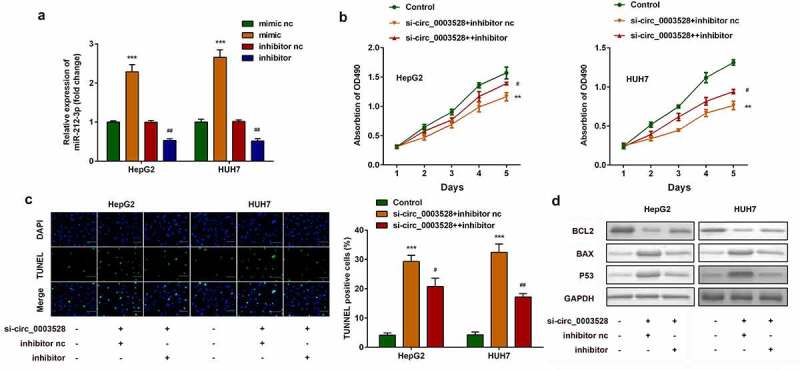
Downregulation of miR-212-3p abrogates the effects of si-circ_0003528 on cell viability and apoptosis of HCC cell lines. (a) miR-212-3p expression was detected by RT-qPCR. (b) Cell viability was detected by MTT assay. (c) The TUNEL assay was performed to evaluate cell apoptosis. (d) Expression of Bcl2, Bax, and p53 proteins.

Next, reverse validation experiments were used to verify the effect of miR-212-3p on circ_0003528. miR-212-3p expression levels sharply decreased in the miR-212-3p inhibitor group and increased in the miR-212-3p mimic group (Figure 4a). Moreover, downregulation of miR-212-3p remarkably abrogated the effects of circ_0003528 knockdown on cell viability and apoptosis of HepG2 and HUH7 cells (Figure 4b-d).

## *XIAP* is a target of miR-212-3p

**Figure 5.**
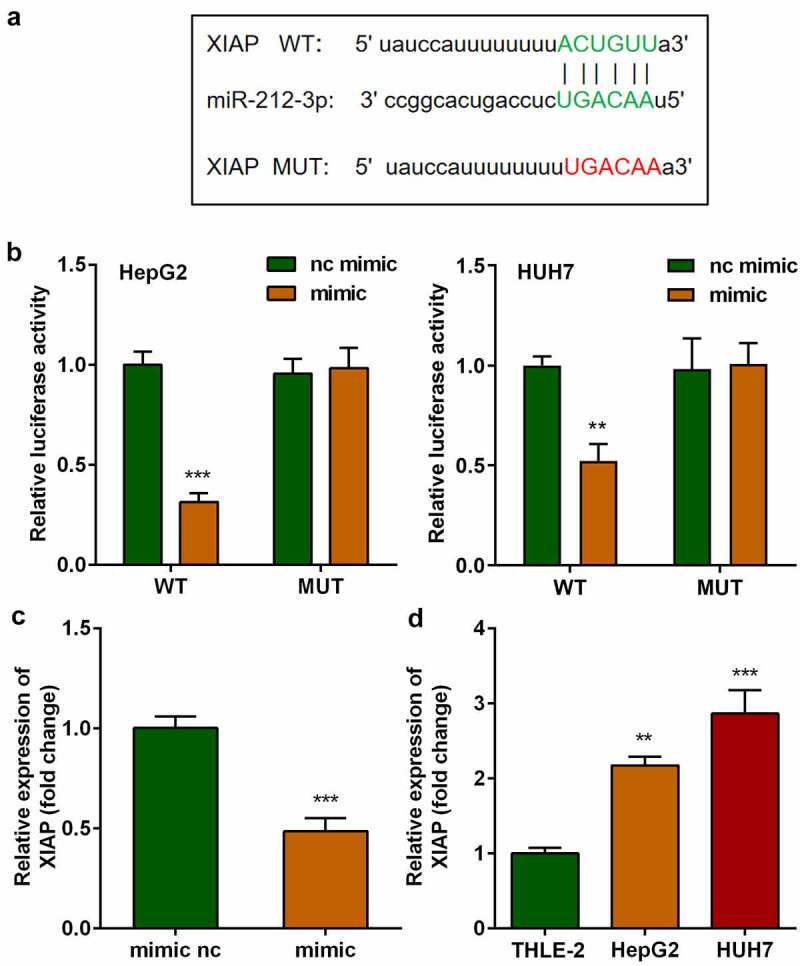
*XIAP* was significantly elevated in HCC and is a target of miR-212-3p. (a) Binding sites between miR-212-3p and *XIAP*. (b) Dual-luciferase reporter assay was performed to confirm the interaction between miR-212-3p and *XIAP* in HCC cells. (c-d) *XIAP* expression.

The targets of miR-212-3p were predicted using the online miRNA target prediction databases, as shown in Figure 5a, miR-212-3p was predicted to bind to *XIAP* by bioinformatic analysis. The binding sites were further verified using a dual-luciferase reporter assay (Figure 5b). The mRNA expression of *XIAP* in cells transfected with the miR-212-3p mimic was markedly decreased compared with that in the nc mimic group (Figure 5c). Moreover, miR-212-3p was found to be overexpressed in HCC cells (Figure 5c-d).

## Upregulation of *XIAP* inhibits the functions of miR-212-3p

**Figure 6.**
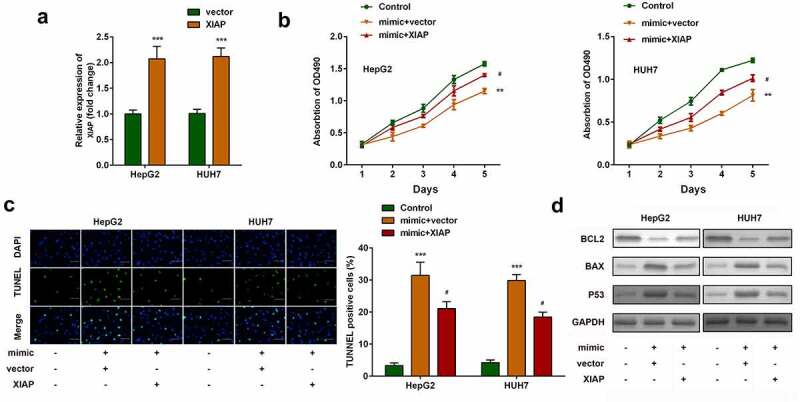
Upregulation of *XIAP* inhibits the effects of miR-212-3p. (a) *XIAP* expression levels were detected by RT-qPCR. (b) Cell viability was detected by MTT assay. (c) The TUNEL assay was performed to measure cell apoptosis. (d) Expression of Bcl2, Bax, and p53 proteins.

The effects of *XIAP* were then investigated. As shown in Figure 6a, *XIAP* expression levels were significantly upregulated in both HepG2 and HUH7 cells transfected with *XIAP* overexpression plasmids. Compared with miR-212-3p-over-expressing cells, overexpression of *XIAP* significantly increased cell viability (Figure 6b) and inhibited apoptosis of HCC cells (Figure 6c-d).

## Knockdown of circ_0003528 inhibited tumor growth of HCC *in vivo*

**Figure 7.**
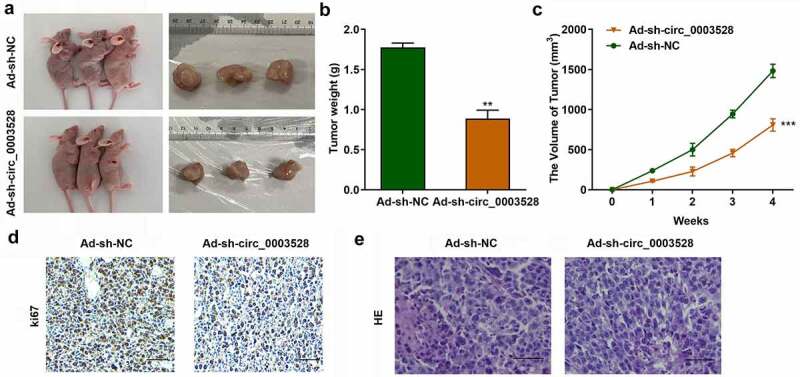
Knockdown of circ_0003528 suppresses tumor growth in vivo. (a) tumor size, (b) tumor volume, and (c) weight of HCC. (d) IHC staining and (e) HE staining for tumor tissues. **p< 0.01, ***p< 0.001, vs. ad-sh-NC group. The scale length is 200 μm.

To verify the function of circ_0003528 in HCC, xenograft assays were used. As shown in Figure 7, circ_0003528 knockdown significantly suppressed tumor size (Figure 7a), weight (Figure 7b), and volume (Figure 7c), suggesting that knockdown of circ_0003528 suppressed tumor growth in HCC. Ki67 protein was located in the nucleus, and IHC was used to detect the expression of Ki67 so as to characterize the tumor formation. Positive staining showed (brown) yellow particles. The results showed that Ki67 expression was positive in most of the transplanted tumor cells in the control group, but the expression of Ki67 protein was lower after transfection with Ad-sh-circ_0003528 than in the control group (Figure 7d). After HE staining, large nuclei, uneven chromatin, and small cytoplasm were observed in the negative control group; however, after transfection with Ad-sh-circ_0003528, the cell morphology was more normal than that of the control group (Figure 7e).

## Discussion

Our study indicated that circ_0003528 was abnormally elevated in HCC, and its inhibition suppressed the aggressiveness of HCC cells. Furthermore, our data demonstrated that circ_0003528 sponges miR-212-3p to regulate *XIAP* expression, thereby manipulating cell fate and promoting tumorigenesis.

A known function of circRNAs to date is that they mainly regulate gene expression by directly or indirectly regulating gene regulators (such as miRNAs) [9,14]. Because of their stable expression in tissues and body fluids, circRNAs are recognized as potential biomarkers of tumors and other disorders and have attracted increased attention [28]. For instance, circRNAs, such as circRNA-5692, circRHOT1, and circRNA cSMARCA5, affect cellular functions, including proliferation, apoptosis, and migration and invasion of HCC cells [10,12,13]. In addition, drug resistance and angiogenesis, which are associated with HCC progression, are regulated by various circRNAs, such as circRNA SORE and circRNA-100338 [Huang and Huang et al., 2020; 29]. Moreover, the β-catenin, mTOR, and MEK/ERK [30–32] pathways modulated by circRNAs have been demonstrated to participate in HCC development. Therefore, it is important to elucidate the regulatory mechanisms of circRNAs in HCC. Our bioinformatics analysis results indicated that circ_0003528 was abnormally overexpressed in HCC tumor tissues, and the expression status of circ_0003528 in HCC cells was consistent with the bioinformatics results. Furthermore, the changes after circ_0003528 inhibition were investigated to evaluate its anti-viability and pro-apoptotic role in HCC. Knockdown of circ_0003528 suppressed tumor growth *in vivo*. Therefore, combined with the gene regulatory function of circRNAs, circ_0003528 was speculated to regulate HCC progression by sponging miRNAs.

Previous studies have revealed that circRNAs participate in various biological processes by sponging miRNAs. In the current study, miR-212-3p was identified as a target miRNA of circ_0003528. According to a recent study, miR-212 can act as a tumor suppressor gene by affecting disparate targets or pathways during cancer initiation, progression, and metastasis [33,34]. miR-212-3p suppresses high-grade serous ovarian cancer development by directly targeting MAP3K3 (Zhang and Zhang et al., 2020). Li et al. [31] found miR-212-3p served as a suppressor of breast cancer carcinogenesis via regulating VEGFA. Likewise, Yuan et al. [35]reported that miR-212-3p had a cancer-inhibiting effect in HCC. These studies revealed that miR-212-3p is an antitumor miRNA. Our data showed that downregulation of miR-212-3p reversed the antitumor effects of si-circ_0003528 in HCC cells, indicating the carcinostatic potential of miR-212-3p, which is in line with results of previous studies [6,35].

*XIAP* is a member of the inhibitor of apoptosis proteins (IAPs) (proteins that prevent the death of host cells) family [36]. IAPs are involved in many aspects of cell death regulation, from inhibiting apoptosis and necrosis to regulating cell cycle and inflammation [37]. Because of their outstanding capacity to regulate cell death along with their high expression in many types of tumor cells, IAPs may be a new target for cancer therapy in the future. Increasing evidence suggests that suppression of *XIAP* can inhibit tumor growth in gastric cancer [38], prostate cancer [39], colon cancer [40], and HCC [41]. In this study, *XIAP* could bind with miR-212-3p and mechanistically antagonize the inhibitory effect of miR-212-3p on carcinogenesis, indicating that *XIAP* is likely to be associated with tumorigenesis and enhance the malignant behavior of HCC cells, which is consistent with the results of 41[41].

## Conclusions

Thus, silencing circ_0003528 alleviated HCC development both *in vitro* and *in vivo*. Mechanistically, downregulation of circ_0003528 functions as a tumor suppressor by sponging miR-212-3p by targeting *XIAP*. Therefore, knockdown of circ_0003528 is a promising curative method for HCC treatment.

## Supplementary Material

Supplemental Material
