# Supplementary figures and images for: Down-regulated HSA_circ_0003528 inhibits hepatocellular carcinoma aggressiveness via the miR-212-3p/XIAP axis
Source: Bioengineered. 2022 Apr 29;13(4):11269–80. doi: 10.1080/21655979.2022.2066046 (PMC9208529; doi:10.1080/21655979.2022.2066046)

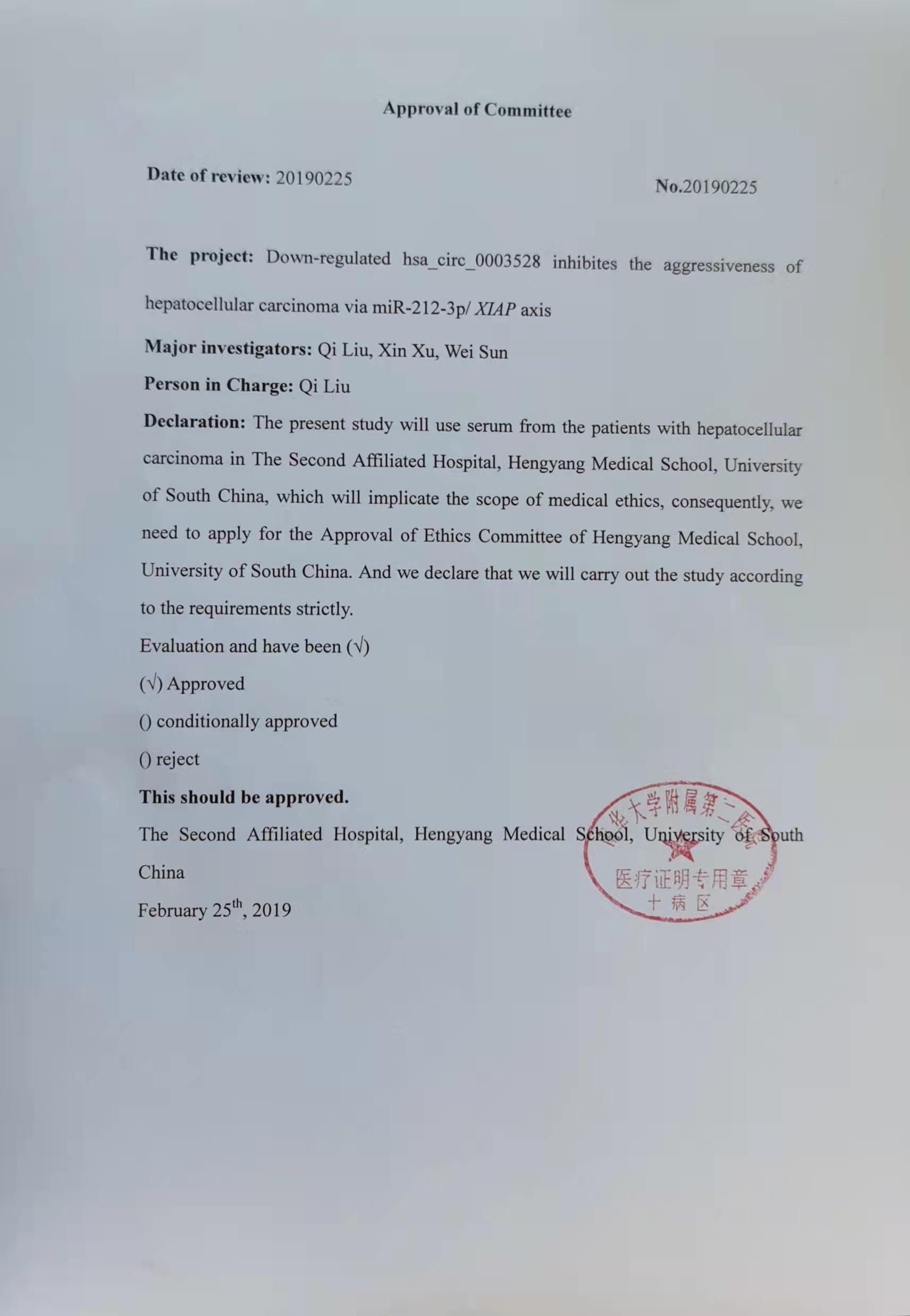

Supplement: Supplemental Material [file KBIE_A_2066046_SM2403.zip › supplementary/ethical approval.jpg]
